# A New Model of a Macular Buckle and a Refined Surgical Technique for the Treatment of Myopic Traction Maculopathy

**DOI:** 10.3390/vision8030042

**Published:** 2024-07-03

**Authors:** Barbara Parolini

**Affiliations:** Eyecare Clinic, Via Cefalonia 70, 25124 Brescia, Italy; parolinibarbara@gmail.com

**Keywords:** myopic traction maculopathy, macular buckle, maculoschisis, macular detachment, high myopia

## Abstract

Myopic traction maculopathy (MTM) affects 20% of eyes with pathologic myopia (PM). The MTM Staging System (MSS), published in 2020, describes the nomenclature of MTM as well as a proposal of pathogenesis, natural evolution, and prognosis. A study of customized treatment for each stage of MTM has been published previously and suggested to treat maculoschisis and detachment by placing a macular buckle (MB) behind the macula to push the sclera towards the retina, selecting pars plana vitrectomy (PPV) only in cases where a macular hole is associated with MTM. We hereby describe a new model of a macular buckle, known as NPB, and an NPB loading device, with the aim to standardize the surgical technique and render it more user friendly, efficient, and safe. Macular buckle is an effective and safe procedure to treat maculoschisis and macular detachment in MTM. We recommend using it as a unique and first-line treatment.

## 1. Introduction

Myopic traction maculopathy (MTM) affects 20% of eyes with pathologic myopia (PM) [1,2].

The MTM Staging System (MSS), published in 2020, describes the nomenclature of MTM as well as a proposal of pathogenesis, natural evolution, and prognosis [3]. The MSS has been internationally validated [4].

**Figure 1:**
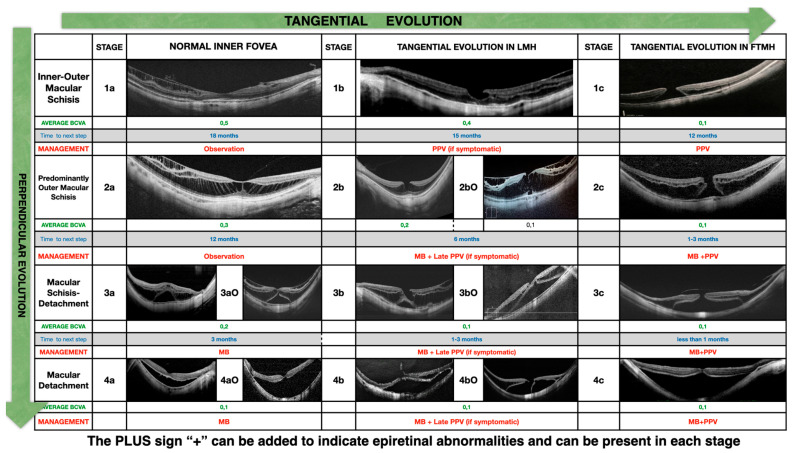
MSS table.

The MSS is summarized in Figure 1.

The four rows represent the evolution of the disease in a direction perpendicular to the retina, starting from inner/outer schisis (stage 1) and evolving to predominantly outer schisis (stage 2), to a combination of schisis and detachment (stage 3), and finally to complete macular detachment (stage 4). When the macula is completely detached, as in stage 4, the schisis in the macula disappears.

The three columns represent the evolution in a direction tangential to the retina and the fovea from a normal foveal profile (stage a) to a inner lamellar macular hole (stage b) to a full-thickness macular hole (stage c).

The presence of an outer lamellar macular hole is marked as a capital O and might occur in stages 2, 3, and 4.

The presence of epiretinal abnormalities of any kind is marked with the sign + (read as “plus”) and might occur in every stage.

A proposal of treatment of MTM, customized per stage, has been published previously [5] and suggested to treat maculoschisis and detachment by placing a macular buckle (MB) behind the macula to push the sclera towards the retina and to treat the holes in stage c with pars plana vitrectomy (PPV) and maneuvers on the ILM.

A previous description of the macular buckle technique [6] and the indications of use [7] has been published. In summary, it was suggested to observe stages 1a and 2a, to perform PPV and maneuvers on the ILM in stages 1b (only in case of severe drop in vision) and 1c, to perform macular buckle surgery in stages 3a, 3b, 4a, and 4b, and to perform combined PPV MB in stages 2c, 3c, and 4c. In the case of combined surgery, it can be chosen to apply a macular buckle as the first and only procedure to treat schisis/detachment of the macula and decide whether to add PPV and maneuvers on the ILM only at a later stage to close the macular hole.

The macular buckle procedure is still considered difficult and associated with intra- and postoperative complications [8,9]. These limitations of macular buckle surgery were due either to previous models of buckle [10,11,12] with materials and shapes prone to induce complications and to surgical techniques that implicated awkward and difficult maneuvers that were finally abandoned.

There is no standardization of the surgical technique nor a well-established sequence of surgical steps, and it does not use widely accepted or commercially available products.

The author of this paper has extensive experience in macular buckle surgery and has personally performed more than 400 procedures using different models of macular buckles and surgical strategies [13]. Having obtained consistent results in terms of efficacy and safety in the treatment of MTM, as previously published, the authors concentrated on the design of a new user-friendly model of a macular buckle and a more standardized repeatable and approachable surgical procedure. A recent publication reported anatomical success in 100% of cases and a significant improvement in vision when compared to the preoperative stage [13].

### 1.1. The New Medical Device—NPB

**Figure 2:**
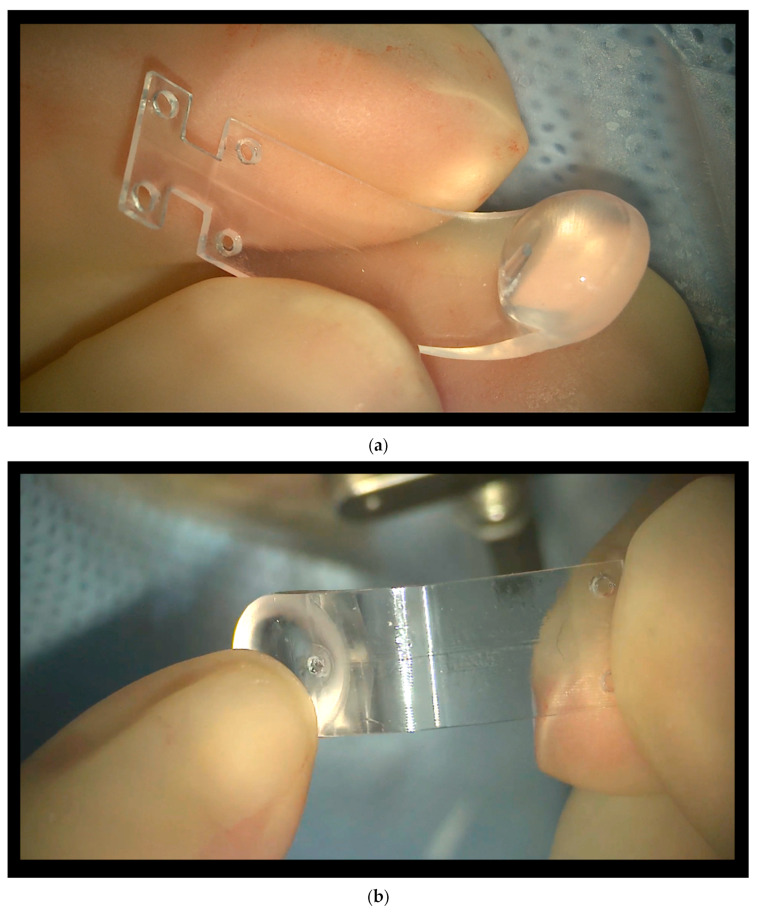
(**a**) The new macular buckle (NPB), internal side. A hand holds the NPB, which has 2 anterior wings with holes. Two more holes are placed 3 mm more posteriorly. The internal side is inserted facing the eyewall. (**b**) The new macular buckle (NPB), external side. The lodge to host the illuminated fiber is shown behind the head of the NPB.

We hereby describe a new model of a macular buckle (Figure 2a,b), known as NPB (AJL, Alava Spain), designed by the author, and a standardized, repeatable surgical technique with the aim to render it more user-friendly, efficient, and safe.

The NPB is a one-piece J-shaped device, made of medical-grade PMMA and covered by a thin biocompatible silicon layer. It can be custom-made, based on the axial length of the eye. The buckle has two main parts, the head and the arm. We call the “inner side of the NPB” the side facing the eyewall when the NPB is inserted. The external part of the buckle faces the orbit.

The head is the buckling side when placed behind the macular sclera. The head has a round base and is a hemispheric shape with a flat surface. The external side of the head of the NPB has a lodge to host an illuminated fiber.

The arm, which has a J shape, serves to position and suture the buckle. The arm is as wide as the head. The anterior side of the arm has two wings with holes to host the anterior sutures. Two more holes are placed 3 mm posteriorly on the arm of the NPB. 

### 1.2. The NPB Loading Device

**Figure 3:**
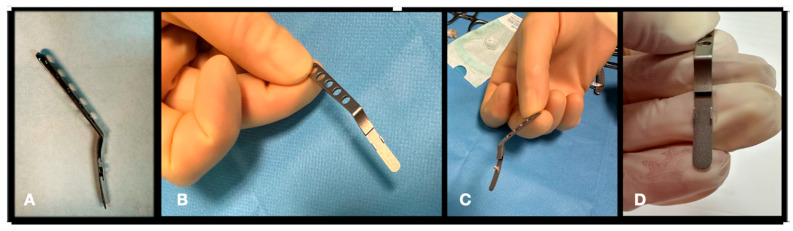
The NPB loading device. (**A**) lateral view. (**B**) Frontal view. (**C**) Grasping the loading device by the handle in lateral view. (**D**) grasping the loading device by the handle in frontal view.

An NPB loading device (Janach, Como, Italy), visible in Figure 3, was also designed to ease the insertion of the NPB loading device; it connects perfectly, matching the NPB arm, and has three main functions:

1. To allow a fluent insertion.
2. To allow a comfortable check of the NPB position by facilitating the dynamic indentation movements.
3. To keep the NPB stable during the suture’s applications.

## 2. Surgical Technique

Surgery may be performed under general or local anesthesia. For local anesthesia, we prefer to combine sedation and sub-Tenon anesthesia with a blunt cannula to avoid the risk of scleral perforation with retro- or parabulbar needle injections. The total amount of anesthesia should not exceed 6 cc in total in order not to raise the intraocular pressure (IOP).

A tutorial video is offered as Supplementary Materials to the present manuscript.

### Surgery Step by Step

**Figure 4:**
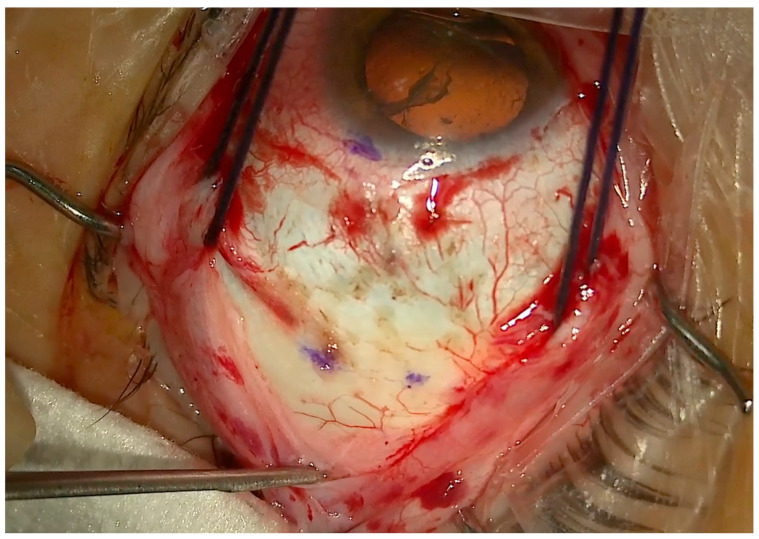
Traction threads placed around the superior and lateral rectus muscles to mobilize the eye and expose the superotemporal quadrant. Three marks are visible to highlight the DLN.

1. Conjuctival and Tenon Separation. The aim of this step is to perfectly expose the sclera in the superotemporal quadrant, preserving the conjunctiva and Tenon for the final suture and making it able to cover the macular buckle with tissue well at the end of surgery.
  - Superior and temporal circumferential conjunctival peritomy is performed in the perilimbal area, preserving the limbus, from 11 to 4 o’clock in the left eye and from 8 to 1 o’clock in the right eye. A radial conjunctival peritomy is advised at the edges of the circumferential peritomy and it should be 2–3 mm at the superonasal edge and 10 mm at the inferotemporal edge.
  - The Tenon is dissected from the sclera, trying to preserve its integrity as much as possible.
  - The sclera in the superotemporal quadrant is freed from bleeding vessels by applying spot diathermy to obtain a clean surface on which to place the NPB.
2. Mobilise the Eye. It is important to be able to mobilize the eye during this type of surgery. There are many methods to reach this goal.
  - The lateral and superior rectus muscles are exposed by at least 4 mm by gently polishing the insertion from the Tenon capsule. A violent and deeper exposure of the muscle might induce an inflammatory reaction with fibrotic scars. A thick traction thread (for example, Vycril 0\0) is placed around the insertion of the lateral rectus muscle and then knotted (Figure 4). The same is performed for the superior rectus muscle. Traction threads around the muscle insertion allow an easier mobilization of the globe without damaging any tissue. This is what we have always used.
  - As an alternative, without exposing the whole muscle insertion, a traction suture can be placed directly into the insertion of the muscles (the superior corner of the lateral and the temporal corner of the superior muscle), but this maneuver could stretch and damage the insertion.
  - One more alternative is to place the suture into the anterior sclera. We do not recommend this if the sclera is thin.

However, it is important to highlight that by using the NPB loading device, step 2 could be totally skipped, because the NPB loading device can be used to mobilize the NPB and the eye itself.

**Figure 5:**
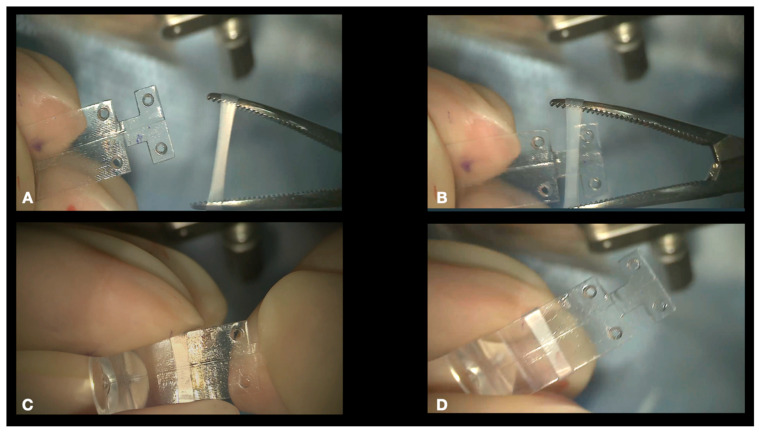
A silicone sleeve is stretched by pean forceps (**A**,**B**) and placed around the NPB arm (**C**,**D**). The final position of the sleeve is roughly in the middle of the length of the arm to host the tip of the NPB loading device and the illuminated fiber.

**Figure 6:**
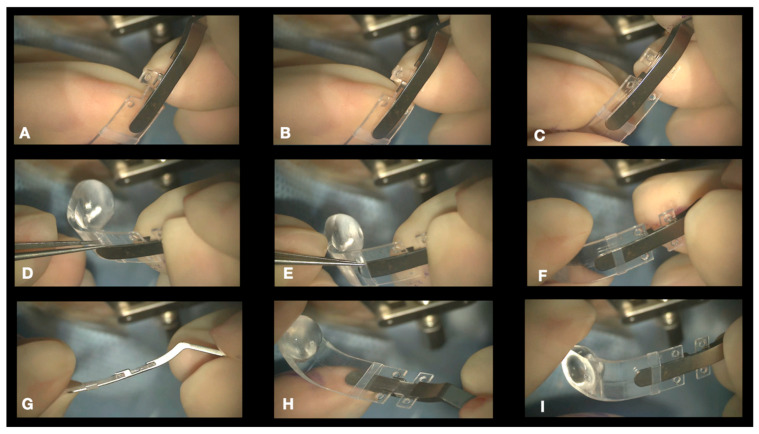
Connection of the NPB loading device to the NPB. The loading device is held by the handle and aligned to the external side of the NPB arm (**A**,**B**). The teeth of the loading device embrace the NPB anterior wings (**C**). The tip of the loading device is inserted into the silicone sleeve (**D**–**F**) with non-toothed forceps. Final visualization of the loading device (**G**–**I**).

**Figure 7:**
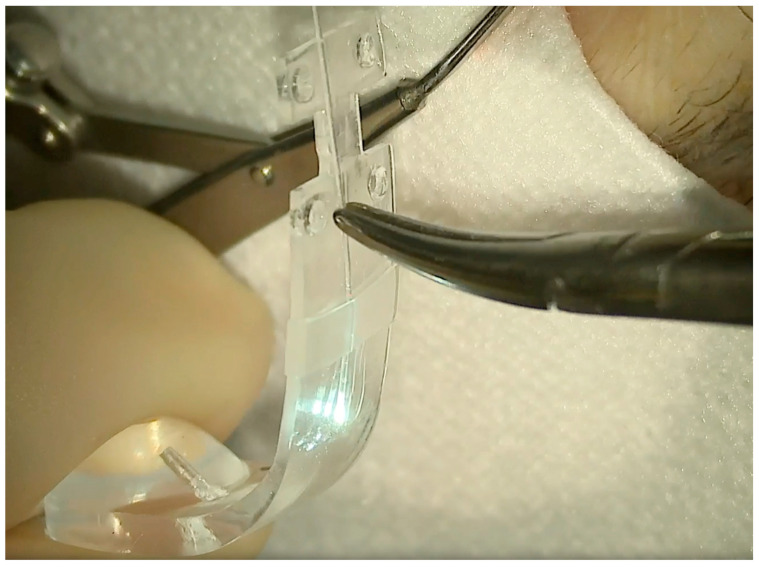
An illuminated fiber is slid onto the external side of the NPB arm through the silicone sleeve and then inserted into the lodge behind the NPB head.

**Figure 8:**
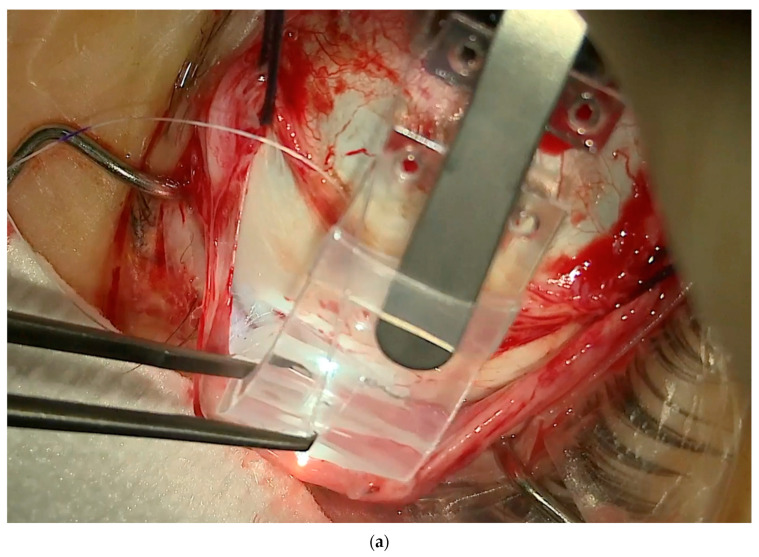

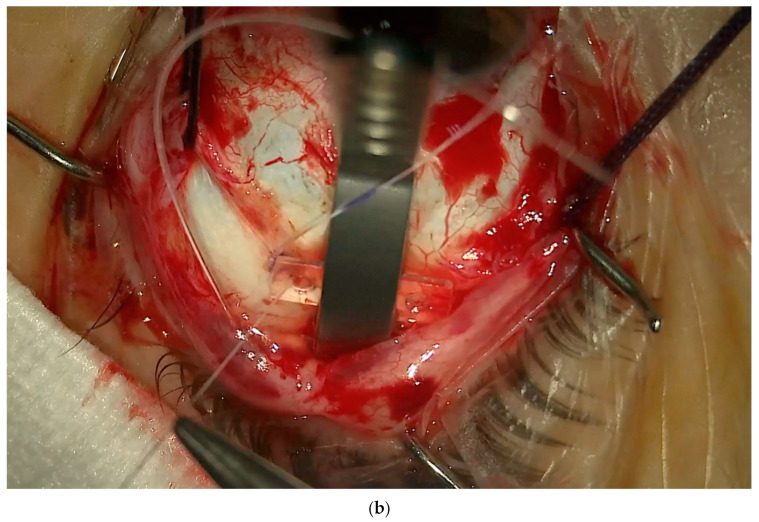
(**a**) The NPB connected to the NPB loading device is inserted into the superotemporal quadrant, pushing the head toward the posterior pole. In this case, an illuminated fiber is loaded too. (**b**) The NPB head slipped behind the macula. One suture is placed into one anterior hole.

**Figure 9:**
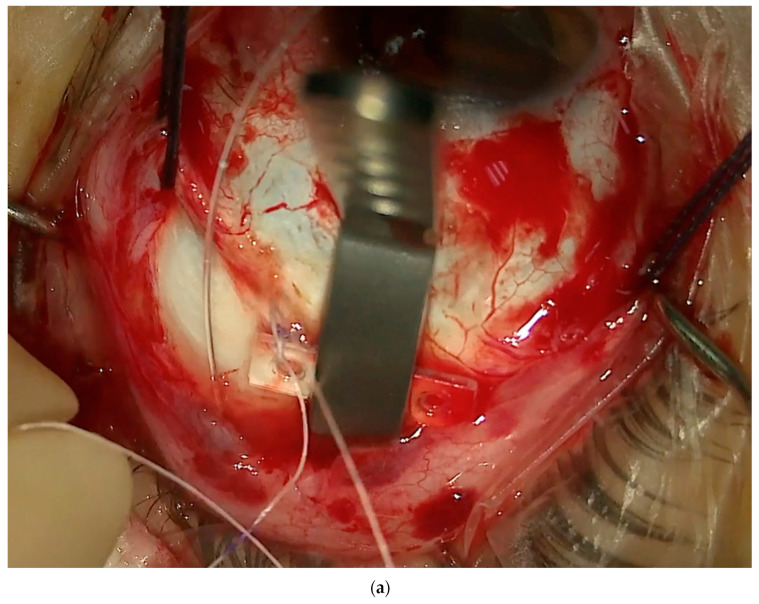

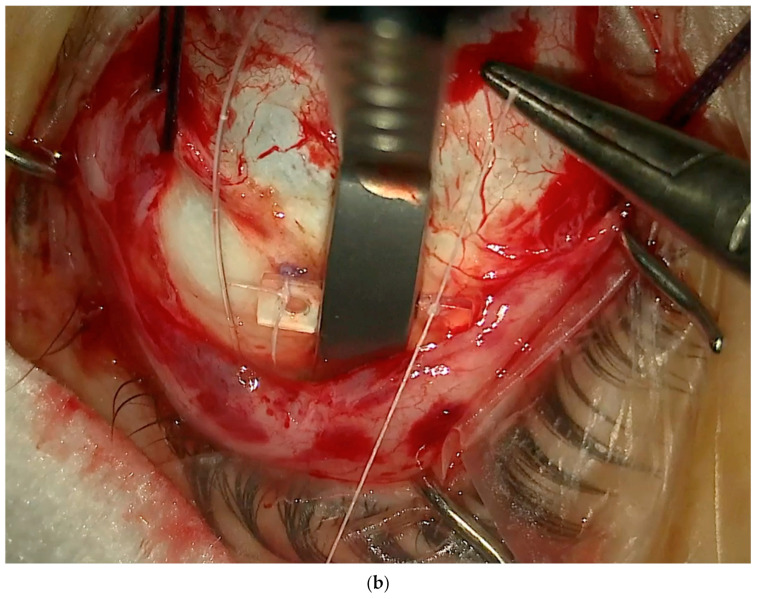
(**a**) One suture secured in the anterior hole. (**b**) Second suture secured in the anterior hole while the NPB is held by the NPB loading device.

**Figure 10:**
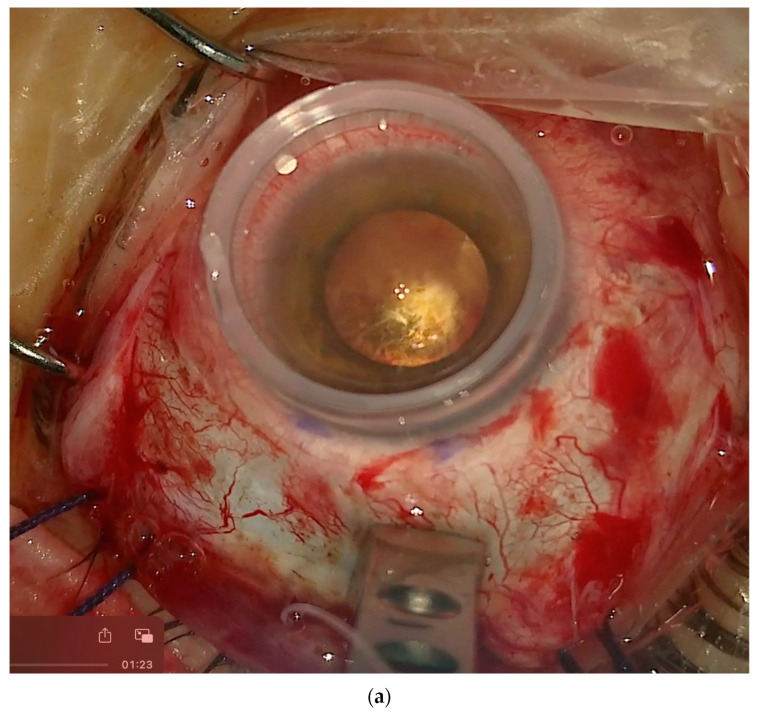

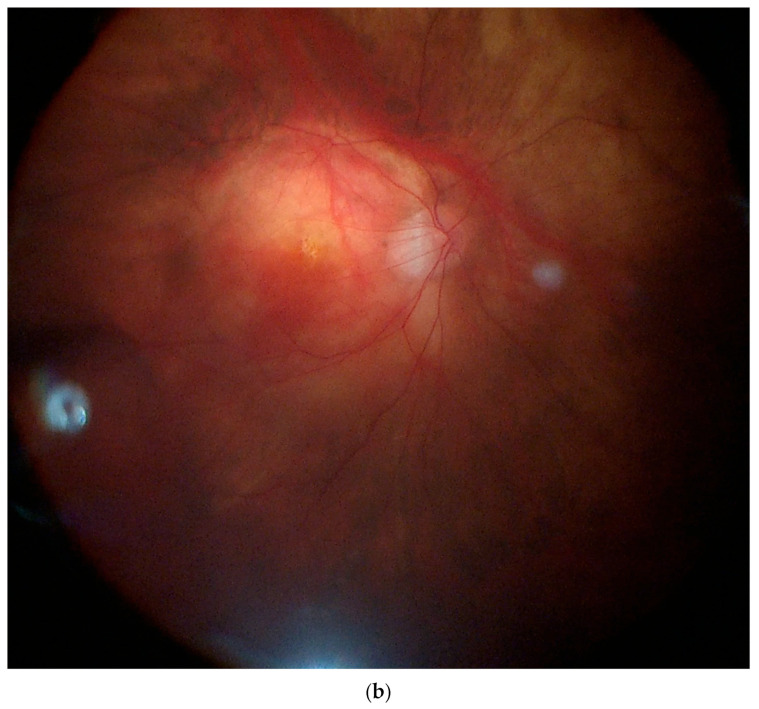
(**a**) View through the microscope and a flat contact lens on the cornea. The macula is highlighted using transillumination. Through the microscope, it is possible to see both the macula and the NPB loading device. (**b**) View through a panoramic widefield viewing system. In this case, we can see the fundus. The macula and the indentation of the buckle are visible due to the transillumination effect. It is not possible to see the NPB simultaneously.

3. Determining the DLN. The suture placement is the most important step of the whole surgery. The distance from limbus–needle (DLN) is marked to indicate where to enter with the needle to place the anterior superior and temporal sutures (Figure 4). Three points are marked with a caliper and staining, ideally marking the three corners of a triangle, with the apex at the limbus. The limbal point is marked at the 2:30 position in the left eye and at the 10:30 position in the right eye. The superior and temporal DLN points are marked according to the nomogram (described below).
4. Preparing the NPB. The NPB can be inserted into the superotemporal quadrant without any preparation. The maneuvers which are described in step 4 are not indispensable but useful to make the procedure easier and safer. Both maneuvers require the use of a silicone sleeve, which is provided with the NPB package. The silicone sleeve can be easily positioned around the arm of the buckle, roughly in the middle of the length of the arm (Figure 5).
  a. Connecting the NPB on the NPD Loading device. We suggest connecting the NPB to the NPB loading device (Figure 6). As an alternative, the NPB can be held with bayoneted forceps, but attention should be taken not to break the NPB. To connect the NPB to the NPB loading device, it is necessary to execute the following steps:
    - Hold the handle of the NPB loading device.
    - Align the tip of the loading device to the external side of the NPB arm.
    - Insert the tip of the loading device into the silicone sleeve with the help of non-toothed forceps.
    - Connect the teeth of the loading device, embracing the NPB anterior wings.
  b. Connecting the Illuminated Fiber to the NPB. A 25-, 27-, or 29-gauge illuminated fiber can be slid onto the external side of the arm through the silicone sleeve and finally inserted into a dedicated lodge under the head of the NPB (Figure 2b and Figure 7). The light helps with the transillumination function, but we observed that surgery can be performed without any light and using only the microscope view.
5. Insertion of the NPB. The NPB is inserted into the superotemporal quadrant by gently sliding and pushing the head, with a rotational movement, first along the eyewall and finally toward and behind the posterior pole (Figure 8a,b). The head is placed under the macula.
6. Superior and Temporal Suture. The sutures are secured into the anterior holes of the NPB arm (Figure 9a,b).
7. Position Check. The position of the NPB is checked through the microscope by moving the NPB with the NPB loading device or with strong forceps (Figure 10a,b). If the location is correct, the surgeon can go to step 8; otherwise, the sutures should be adjusted until the buckle is well centered.
8. Disconnecting the NPB Loading Device From the NPB. If the NPB loading device was used, at the end of the procedure, it must be carefully disconnected from the NPB. We advise to keep the NPB’s most anterior wing steady with non-toothed forceps and to gently elevate it by 1mm and pull the NPB loading device out.
9. Ending the Procedure. The traction sutures around the muscles are removed, and the Tenon is dried and placed over the buckle, suturing one spot in the inferotemporal quadrant. Then, the conjunctiva is dried and placed with care over the buckle and the Tenon and sutured with reabsorbable sutures (Vycril 7/0). Ideally, one suture is placed at the level of the superonasal peritomy and three sutures are placed at the inferoteporal radial peritomy.

## 3. Viewing the Maneuvers

All the maneuvers can be made under the microscope in two different modalities: 1. with a flat contact lens over the cornea or 2. with a panoramic widefield viewing system (PVS). 

The advantage of the flat contact lens (Figure 10a) is that, through the microscope, it is possible to check simultaneously both the location of the head of the buckle under the macula through the cornea and the location of the arm of the buckle over the sclera. The disadvantage is the small field of view over the macular area. The contact lens is naturally dislodged while moving the eye nasally to place the sutures. Then, the lens should be cleaned and repositioned to check the location of the buckle. 

The advantage of the PVS (Figure 10b) is that it offers a widefield view. Therefore, the location of the fovea is easier to identify, and it is possible to simultaneously see the location of the macular buckle under the fovea and the effect of the arm of the buckle on the periphery. The disadvantages of the PVS are double-fold. First, the view though a PVS requires the placement of an illuminated fiber in the pars plana or transillumination, and secondly, the eye needs to be kept straight and parallel to the microscope to locate the buckle, while to suture the NPB, the PVS must be removed, and the eye should be moved nasally to expose the temporal quadrant. This is more time-consuming.

With the new NPB and with a contact flat lens, these procedures are faster and easier and the surgeon can perform every maneuver independently.

## 4. Nomogram

To further ease and standardize the surgical procedure, we developed a nomogram that predicts where to place the sutures based on the axial length of the eye. Our variable was the distance between the limbus and the superior and temporal points of insertion of the needle. We called this variable “distance from limbus–needle” (DLN). 

The limbal point should be marked in the middle of the superotemporal quadrant (at the 2:30 position for the left eye and at the 10:30 position for the right eye).

We collected data from 40 eyes with a range of axial length between 27.94 and 35.65 mm, using, in every case, an NPB with a total length of 23.6 mm. The table with the collection of superior and temporal DLNs based on the axial length is attached in Table 1. Knowing the DLN allows us to speed up the procedure. 

A new nomogram was elaborated for obtaining NPBs of different sizes with the final goal of obtaining a fixed DLN that is always 12 ± 2 mm from the limbus. In fact, with longer DLNs, the suture is difficult to place and deep into the orbit, while a shorter DLN increases the risk of secondary buckle exposure and extrusion through the conjunctiva. The ideal DLN is in the range of 11 to 13 mm from the limbus. 

The NPB’s custom size can be required to the company based on the axial length.

### 4.1. Further Notes on The Technique

It is possible to insert a light in pars plana, but this maneuver should be reserved only in cases of macular buckle viewed through a PVS or if the buckle surgery is performed in combination with pars plana vitrectomy. If only the macular buckling is performed, we suggest not to change the IOP and therefore avoid paracenteses in the anterior chamber and sclerotomies.

The sutures should be non-reabsorbable and have a spatulated needle. We have always used T-cron 6/0. The two anterior sutures guarantee the position of the buckle. The two posterior sutures offer a higher buckling effect and more stability to prevent dislocation and tilting. 

It is important to avoid excessive indentation of the sclera. The final profile of the retina and the sclera should be ideally as flat and horizontal as possible, resembling a non-myopic macula.

### 4.2. Complications of Macular Buckle and How to Avoid Them

Possible complications of macular buckle were studied:

- Superficial extrusion of the lateral arm of the macular buckle (5%), which manifests as a late complication, from 6 months to years postoperatively.
  ○ Tips to avoid it: Keep an ideal position of the DLN of 11 mm or more by calculating the correct length of the NPB based on the axial length.
- Diplopia (5%), mostly in the form of hypotropia.
  ○ Tips to avoid it: Advise the patient to move the eye in every direction while keeping the head still multiple times in the next two weeks after surgery. This helps to avoid fibrosis around the buckle in the orbit. Smaller NPBs are under study.
- Peripheral choroidal hemorrhage (0.5%), but only 0.05% were submacular.
  ○ ○ Tips to avoid it: Keep the IOP stable during surgery, avoiding paracentesis or opening of the vitreous chamber. Try to avoid multiple maneuvers over the sclera to insert the buckle. This will help to not stress the vortex veins with scleral massage.

**Figure 11:**
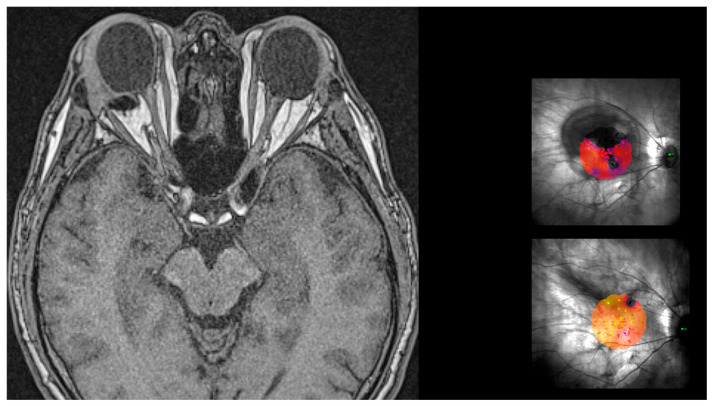
MRI of brain and orbits showing the shape of the head of the macular buckle behind the right eye. The buckle does not touch the optic nerve. This patient was operated on 11 years ago. The microperimetry on top shows the large scotoma prior to surgery and the one on the bottom shows the complete resolution of the scotoma after surgery.

Trauma to the optic nerve was never observed. Eyes were followed with magnetic resonance imaging (MRI) of the orbits. MRI shows the profile of the buckle and the untouched optic nerve (Figure 11).

Recently, we completed a study to evaluate the rate of progression of myopic macular atrophy and choroidal neovascularization (CNV) after macular buckle. The study compared the operated eye versus the contralateral non-operated eye with a similar degree of high myopia. The study showed a similar rate of progression of atrophy in both eyes and a higher rate of choroidal neovascularization in the contralateral eye [14].

## 5. Conclusions

Macular buckle is an effective and safe procedure to treat maculoschisis and macular detachment in MTM. New interest in this surgical technique was demonstrated by different authors [15,16].

The surgical technique, as described in this paper, is standardized and repeatable.

We recommend using it as a unique and first-line treatment in stage 3a, 3b, 4a, and 4b MTM.

We recommend using macular buckle prior to or in combination with PPV and ILM flap in stage 2c, 3c, and 4c MTM and in all cases of MTM after failure of primary PPV.

The new NPB with the NPB loading device, the new surgical procedure, and the nomogram may help surgeons by providing a standardized, easy, and repeatable procedure.

## Figures and Tables

**Table 1:** Preliminary nomogram to predict DLN (based on an NPB with 23.6 mm length).

AL Pre	Superior DLN	Temporal DLN
28.06	11.00	9.00
28.61	10.00	9.00
29.02	11.50	9.00
29.23	12.00	8.00
29.37	9.50	9.00
29.6	9.50	9.00
29.78	10.00	10.00
30.03	12.00	9.00
30.09	11.50	10.00
30.36	12.00	11.00
30.62	10.00	8.50
30.78	11.50	9.00
30.85	11.50	10.50
31.03	11.00	10.50
31.17	12.00	10.00
31.49	12.00	11.00
31.52	11.50	11.50
31.55	12.50	12.00
31.50	12.50	11.00
31.62	11.50	11.50
31.81	12.00	10.00
31.90	13.00	12.50
31.18	13.50	12.00
32.81	13.00	13.00
32.91	13.50	13.00
33.89	14.50	14.00
34.31	14.00	14.00
34.49	15.00	14.00
34.51	14.00	14.00
34.85	14.00	12.50
35.39	15.00	14.00
35.65	16.50	15.00

## Data Availability

Data are unavailable due to privacy restrictions.

## Supplementary Materials

The following supporting information can be downloaded at: https://www.mdpi.com/article/10.3390/vision8030042/s1.

## References

[1] Panozzo G., Mercanti A. (2004). Optical coherence tomography findings in myopic traction maculopathy. Arch. Ophthalmol..

[2] Baba T., Ohno-Matsui K., Futagami S., Yoshida T., Yasuzumi K., Kojima A., Tokoro T., Mochizuki M. (2003). Prevalence and characteristics of foveal retinal detachment without macular hole in high myopia. Am. J. Ophthalmol..

[3] Parolini B., Palmieri M., Finzi A., Besozzi G., Lucente A., Nava U., Pinackatt S., Adelman R., Frisina R. (2021). The new Myopic Traction Maculopathy Staging System. Eur. J. Ophthalmol..

[4] Parolini B., Arevalo J.F., Hassan T., Kaiser P., Rezaei K.A., Singh R., Sakamoto T., Rocha J., Frisina R. (2023). International Validation of Myopic Traction Maculopathy Staging System. Ophthalmic Surg. Lasers Imaging Retin..

[5] Parolini B., Palmieri M., Finzi A., Frisina R. (2021). Proposal for the management of myopic traction maculopathy based on the new MTM staging system. Eur. J. Ophthalmol..

[6] Parolini B., Frisina R., Pinackatt S., Mete M. (2013). A New L-Shaped Design of Macular Buckle to Support a Posterior Staphyloma in High Myopia. Retina.

[7] Parolini B., Frisina R., Pinackatt S., Gasparotti R., Gatti E., Baldi A., Penzani R., Lucente A., Semeraro F. (2015). Indications and results of a new l-shaped macular buckle to support a posterior staphyloma in high myopia. Retina.

[8] Mateo C., Burés-Jelstrup A. (2016). Macular buckling with ANDO PLOMBE may increase choroidal thickness and mimic serous retinal detachment seen in the tilted disk syndrome. Retin. Cases Brief. Rep..

[9] Alkabes M., Mateo C. (2018). Macular buckle technique in myopic traction maculopathy: A 16-year review of the literature and a comparison with vitreous surgery. Graefe’s Arch. Clin. Exp. Ophthalmol..

[10] Tanaka T., Ando F.U. (2005). Episcleral macular buckling by semirigid shaped-rod exoplant for recurrent retinal detachment with macular hole in highly myopic eyes. Retina.

[11] Stirpe M., Ripandelli G., Rossi T., Cacciamani A., Orciuolo M. (2012). A new adjustable macular buckle designed for highly myopic eyes. Retina.

[12] Wu P.C., Sheu J.J., Chen Y.H., Chen Y.J., Chen C.H., Lee J.J., Huang C.L., Chen C.T., Kuo H.K. (2017). Gore-tex vascular graft for macular buckling in high myopia eyes. Retina.

[13] Ripa M., Motta L., Matello V., Frisina R., Parolini B. (2024). Long-Term results of macular buckle for MTM stage 3–4 With maculoschisis and macular detachment without and with lamellar macular hole. Eur. J. Ophthalmol..

[14] Parolini B., Padrón J.F.R., Lopes E., Matello V., Crincoli E. (2022). Evaluation of macular atrophy in patients treated with macular buckle for myopic traction maculopathy (MTM): Mid and long-term follow-up. Retina.

[15] Kortuem F.C., Ziemssen F., Neubauer J., Bartz-Schmidt K.U., Dimopoulos S. (2022). Introducing a customized low-cost macular buckle. Retina.

[16] Akduman L. (2024). A titanium macular buckle implant designed for an easy placement in myopic macular holes. Retin. Cases Brief Rep..

